# Epigenetic Inhibitor 5-Azacytidine Triggers DIM-2/DIM-5-Dependent Mutagenesis in H3K9me3-Enriched Regions of *Neurospora crassa*

**DOI:** 10.3390/jof12050304

**Published:** 2026-04-22

**Authors:** Ruonan Yao, Jingxuan Chen, Huawei Tan, Yile Sun, Sihai Yang, Long Wang, Ju Huang, Xiaohui Zhang

**Affiliations:** 1 State Key Laboratory of Pharmaceutical Biotechnology, School of Life Sciences, Nanjing University, Nanjing 210023, China; 2 Co-Innovation Center for Sustainable Forestry in Southern China, Nanjing Forestry University, Nanjing 210037, China; 3 State Key Laboratory of Crop Genetics and Germplasm Enhancement, Bioinformatics Center, Academy for Advanced Interdisciplinary Studies, Nanjing Agricultural University, Nanjing 210095, China

**Keywords:** *Neurospora crassa*, 5-azacytidine, mutation, epigenetic, DIM-5, DIM-2

## Abstract

The DNA methyltransferases inhibitor 5-azacytidine (5AzC), clinically used to treat hematopoietic malignancies, can elevate genomic mutational burden, raising safety concerns. To define the epigenetic specificity and mutagenic consequences of 5AzC, we performed multi-omics analyses in *Neurospora crassa*. Our data showed that 5AzC caused a non-selective, genome-wide reduction in both 5-methylcytosine (5mC; ~50% decrease) and the heterochromatin mark H3K9me3 (~65% decrease), indicating broad off-target demethylation that may transiently benefit therapy yet compromise genome stability. Whole-genome sequencing (WGS) revealed a ~290-fold increase in mutation rate under 5AzC, with a pronounced C->G transversion bias, a spectrum typically associated with higher functional burden. Strikingly, 5AzC-induced mutations were enriched in H3K9me3-marked domains, particularly pericentromeric regions characterized by low 5mC but high H3K9me3. Genetic analyses showed that the loss of DNA methyltransferase DIM-2 reduced 5AzC-induced mutations by ~64%, while individual or combined knockout of the histone methyltransferase DIM-5 with DIM-2 led to an 85% reduction. Thus, mutagenesis was markedly amplified by DIM-2 and DIM-5, with DIM-2 activity dependent on DIM-5. Collectively, DIM-2 and DIM-5 accounted for nearly all A/T-site and ~80% of G/C-site mutations. These results reveal that 5AzC drives genome-wide loss of 5mC and H3K9me3, with mutagenesis preferentially targeting H3K9me3-enriched regions via DIM-2 and DIM-5. This work clarifies a mechanistic basis for 5AzC-associated genomic risk and highlights strategies for next-generation epigenetic therapies that preserve heterochromatin integrity while minimizing mutational load.

## 1. Introduction

DNA 5-methylcytosine (5mC) and histone H3 lysine 9 trimethylation (H3K9me3) are two of the most prevalent repressive epigenetic modifications in human cells and are widely conserved across diverse organisms [1,2,3]. These marks, deposited by DNA methyltransferases (DNMTs) and histone lysine methyltransferases (KMTs), are relatively stable through cell divisions [4,5]. Their regulation is critical for numerous cellular processes, including silencing of repetitive elements, repression of developmental genes, DNA repair, and cell differentiation [6,7,8]. Cancer development is often accompanied by both genetic mutations and epigenetic aberrations [9,10]. Promoter hypermethylation and H3K9me3 enrichment frequently silence tumor suppressor genes, thereby promoting tumorigenesis [9,11]. DNMT inhibitors (DNMTis), which induce DNA demethylation and reactivation of silenced genes, have shown therapeutic potential [12,13].

5-azacytidine (5AzC) was the first DNMTi approved by FDA for the treatment of hematopoietic malignancies, and remains in clinical use to prolong patient survival [14,15]. Its efficacy against solid tumors is under investigation in clinical trials [16]. Mechanistically, 5AzC is converted into a DNA-incorporated form that covalently traps DNMTs, leading to their degradation and subsequent DNA demethylation [17,18]. Beyond its effect on DNA methylation, 5AzC also affects the repressive histone marks H3K9me3, resulting in reduced KMT activity and H3K9me3 abundance, which in turn facilitates gene reactivation [19,20,21]. Current research has largely focused on locus-specific effects of 5AzC on DNA demethylation and H3K9me3 suppression, particularly at tumor suppressor genes, whereas its genome-wide epigenetic effects remain poorly characterized. Notably, 5AzC-induced hypomethylation can also activate inhibitory checkpoint molecules, potentially leading to therapeutic resistance and reduced survival, while activation of endogenous retroviruses (ERVs) may contribute both to antitumor effects and to genomic instability [16,22,23,24,25]. Previous studies have shown that 5AzC is toxic to normal cells through DNA demethylation and/or H3K9me3 reduction, causing reproductive defects [26,27]. Given the potentially nonspecific nature of 5AzC-induced gene reactivation, a systematic genome-wide analysis of its effects on DNA demethylation and H3K9me3 is warranted.

In addition to epigenetic suppression, 5AzC can elevate mutational burden in malignant clones, including in key genes such as *TP53*, *TET2*, *DNMT3A*, and *RUNX1* [28,29], which may reduce remission rates and contribute to relapse [30,31,32,33,34]. It also acts as a viral mutagen, inducing G->C transversions and lethal mutagenesis in HIV-1 [35]. Previous studies of 5AzC mutagenesis, largely confined to reporter genes in mice and bacteria, have revealed a conserved C:G->G:C transversion bias [36,37]. Since epigenetic aberrations can drive genomic instability and are linked to mutational processes [38,39], it is important to determine whether 5AzC-induced mutagenesis is associated with its suppression of DNA methylation and H3K9me3. However, many commonly used model eukaryotes (*Caenorhabditis elegans*, *Saccharomyces cerevisiae*, and *Schizosaccharomyces pombe*) lack detectable DNA methylation, making them unsuitable for such studies [40,41].

*Neurospora crassa*, a filamentous fungus, is an ideal model for genetic and epigenetic studies owing to its short life cycle and well-characterized genetic background [40,42]. Unlike in animals and plants, where DNA methylation and H3K9me3 are essential, these epigenetic modifications are dispensable in *N. crassa* [1,2,43,44,45]. Moreover, DNA methylation and H3K9me3 in *N. crassa* are catalyzed by single enzymes, DIM-2 (a DNMT) and DIM-5 (a KMT), respectively, via a streamlined H3K9me3-HP1-DIM-2 pathway [43,46,47]. This simplified system allows for precise dissection of DNA methylation- and H3K9me3- dependent mechanisms without the functional redundancy encountered in higher eukaryotes [1,2]. Additionally, the compact genome of *N. crassa* makes it highly suitable for mutation studies [48]. Collectively, these features position *N. crassa* as a powerful model for elucidating the mechanistic relationship between 5AzC-induced hypomethylation and mutagenesis.

In this study, WGBS and H3K9me3 ChIP-seq revealed that 5AzC acts as a dual inhibitor in *N. crassa*, simultaneously suppressing both DNA methylation and H3K9me3 on a genome-wide scale, with H3K9me3 suppression occurring independently of 5mC loss. WGS data further showed that 5AzC induces strong mutagenesis, with conserved C->G transversions particularly enriched in NCT/NCG contexts, and these mutations were functionally deleterious. Integrative analysis of WGBS, ChIP-seq and WGS data demonstrated that 5AzC preferentially targets H3K9me3-marked regions, especially pericentromeric regions characterized by low 5mC but high H3K9me3 levels. This mutagenic effect was attenuated in the absence of DIM-2 and DIM-5, the absence of DIM-2 reduced mutations by 64%, while individual or combined knockout of DIM-5 with DIM-2 decreased mutations by 85%. This demonstrates that DIM-5 alone maintains a considerable capacity to amplify this mutagenicity, while DIM-2’s enhancement of 5AzC-induced mutagenicity depends on DIM-5. Overall, A/T-site mutations and ~80% G/C-site mutations were mediated by DIM-2 and DIM-5. These results indicate that, in contrast to the genome-wide epigenetic suppression induced by 5-AzC through DNMT and KMT degradation, the presence of these proteins markedly amplifies its mutagenic effects. Insights from this simple system may inform further optimization of 5AzC and guide strategies for drug combinations and relapse prevention in anticancer therapy.

## 2. Materials and Methods

### 2.1. Strains and Reagents

The *ku70^RIP^* strain was a gift from Prof. Qun He, College of Biological Sciences, China Agricultural University, China. This strain exhibits significantly enhanced gene knockout efficiency due to the absence of the Ku70 protein. It was used for creating the *dim-2* or/and *dim-5* mutants (*dim-2*^Δ^, *dim-5*^Δ^, and *dim-2*^Δ^; *dim-5*^Δ^) by disrupting the reading frame through homologous recombination, following a previously described protocol (Figure S1) [49]. All primers used in this study are listed in Table S1. Given that all knockout mutants were constructed on the basis of the *ku70^RIP^* genetic background, throughout this study, the *ku70^RIP^* strain is referred to as the wild-type (WT) strain. To investigate the demethylation and mutagenic effect of 5-azacytidine (5AzC), all strains were grown at 32 °C in Vogel’s medium with or without 5AzC treatment [50]. Previous studies have shown that higher concentrations (e.g., 300 µM) affect *N. crassa* growth (e.g., increased conidiation), whereas 30 µM does not influence conidiation or growth [51]. To minimize growth-related experimental variability, a concentration of 30 µM was selected as the working concentration for 5AzC in this study. A sterile membrane-filtered solution of 5AzC was added to the melted Vogel’s medium to a final concentration of 30 µM. 5AzC was purchased from Sigma-Aldrich (St. Louis, MO, USA), dissolved in dimethyl sulfoxide (DMSO), and stored at −20 °C. A sterile membrane-filtered solution of 5AzC was added to the melted Vogel’s medium to a final concentration of 30 µM. 5AzC was purchased from Sigma-Aldrich, dissolved in dimethyl sulfoxide (DMSO), and stored at −20 °C.

### 2.2. Whole-Genome Bisulfite Sequencing (WGBS) and Calculation of DNA Methylation Level

The strains cultured at 32 °C in Vogel’s medium (with or without 5AzC) for about 36 h, DNA was extracted from the tissues for WGBS. We extracted DNA from the samples using phenol–chloroform extraction and further purified it via isoamyl alcohol precipitation. DNA libraries were prepared with Illumina TruSeq Methylation kit (Illumina, Inc., San Diego, CA, USA) according to the manufacturer’s instructions. Sequencing was performed on Illumina HiSeq 2500 system (Illumina, Inc., San Diego, CA, USA) at BGI Genomics (Shenzhen, China) to generate 150 bp paired-end reads. The methyl-seq data were processed using the Bismark tool (v0.22.1) [52]. The raw reads were mapped to the NC12 reference genome (NCBI accession number GCF_000182925.2) using Bowtie2 (v2.2.6), and bismark_methylation_extractor was used to extract DNA methylation data of Cytosines [52]. To compare DNA methylation differences between samples, DNA methylation levels were calculated with 200bp sliding windows. The methylation level of a specific window is calculated as DNA methylation level = methylated Cs/(methylated Cs + unmethylated Cs).

### 2.3. ChIP-Seq and Data Analysis

The strains were cultured in Vogel’s minimal medium (with or without 5AzC) containing 2% glucose at 32 °C for about 36 h with shaking at 150 rpm. Chromatin immunoprecipitation (ChIP) was performed on tissues using the previously described protocol to obtain immunoprecipitated DNA [53]. For ChIP-seq, the libraries were prepared using 10 ng of immunoprecipitated DNA following the instructions supplied with VAHTS Universal DNA Library Prep Kit (cat. no. ND607). Sequencing was performed on Illumina NovaSeq × Plus system (Illumina, Inc., San Diego, CA, USA at Annoroad (Beijing, China). Adapters and low-quality base calls of ChIP-Seq reads were first removed by Trim Galore (v0.6.10) with the parameters “--quality 20 --length 20” [54]. Subsequently, the processed reads were mapped to the NC12 reference genome with bowtie2 (v2.2.6) with default parameters [55]. ChIP-Seq peaks were called with MACS2 (version 2.2.7.1) with the parameters “--qvalue 0.05 --nomodel --SPMR --bdg --broad” over input files [56]. Peaks less than 2 kb in length were filtered, and peaks with intervals less than 2 kb after filtering were merged into blocks. Normalized BigWig (bw) coverage files were generated using bamCompare (deepTools v3.5.1) with ratio operation and read count scaling [57]. These BigWig files were subsequently converted to bedGraph files. To quantify signals across genomic regions, we employed Bedtools (v2.30.0) map function to calculate average signal intensities within defined genomic windows [58]. For cross-sample comparisons, signals were normalized using a two-step procedure to control for variation in background signal intensities: (1) normalization by total read counts during BigWig generation, followed by (2) scaling based on the median signal intensity of non-peak control regions. The signal quantification results were then integrated with genomic annotation (NC12: GCF_000182925.2) for downstream biological interpretation.

### 2.4. Mutation Accumulation (MA) Experiment

We used the four strains as ancestors for the MA experiment, including WT, *dim-2*^Δ^, *dim-5*^Δ^, and *dim-2*^Δ^; *dim-5*^Δ^ strains. The experiment started by randomly picking a single colony from a Vogel’s medium for ancestors and transferring that colony into another Vogel’s medium. Mediums were incubated at 32 °C for about 60 h to allow the mycelium to grow and make conidia. Then we picked small amount of conidia with a loop into a tube with 200 µL of 0.01% Tween-80 and pipetted 20 µL of this conidial suspension into a 50 µL ddH_2_O droplet on a Vogel’s medium and spread it. We incubated the mediums at 32 °C for ~12 h and randomly picked a single colony to transfer into another Vogel’s medium under a stereomicroscope, and the Vogel’s medium incubated at 32 °C for ~60 h to establish the MA lines. The MA lines were transferred the same way, so that a single colony was always picked randomly from a Vogel’s medium to propagate the MA line. Each transfer cycle, comprising colony generation (~12 h) and mycelial cultures (~60 h), took approximately 3 days to complete.

A total of 35 MA lines were cultured in 5AzC-free medium: 20 derived from the WT, and 5 from each of *dim-2*^Δ^, *dim-5*^Δ^, and *dim-2*^Δ^; *dim-5*^Δ^ mutants. Additionally, 15 MA lines were cultured in 5AzC-supplented medium, comprising 6 from the WT and 3 from each of *dim-2*^Δ^, *dim-5*^Δ^, and *dim-2*^Δ^; *dim-5*^Δ^ mutants. The MA lines of WT, *dim-2*^Δ^, and *dim-5*^Δ^ were cultured for 18 days with 6 transfers (with or without 5AzC treatment), and *dim-2*^Δ^; *dim-5*^Δ^ were cultured for 25 days with 8 transfers (with or without 5AzC treatment).

### 2.5. Whole Genome Resequencing (WGS) and Identification of Mutations

Genomic DNA was extracted from both MA lines and the ancestor strains using the aforementioned protocol. Whole genome resequencing was performed at BGI Genomics (Shenzhen, China)) on an Illumina platform (Illumina, Inc., San Diego, CA, USA) with paired-end 150 bp libraries. On average, each sample was sequenced to a depth of 63× with 97% of the genome covered (Supplementary File S2. Datasheet S1).

Mutations calling was conducted following previously reported pipelines [59,60,61,62] using scripts stored in https://github.com/wl13/BioPipelines, accessed on 20 April 2026. Briefly, cleaned reads were obtained by removing low-quality reads with Q20 < 0.9 using Trimmomatic v.0.39 [63], and were then mapped to the NC12 reference genome with BWA aligner [64]. The resultant sequence alignment/map format (SAM) files were sorted and marked for duplicate reads using SortSam and MarkDuplicates in Picard tools (version 1.114). Raw variants were identified using the Haplotype Caller from Genome Analysis Toolkit (GATK) [65]. Variants that passed the following filters were considered as candidate mutations: (1) For each variant site both ancestor strains and MA lines should have a ≥5 read depth. (2) Both heterozygous and homozygous sites were included. Variants with minimum number of 5 supporting reads in a given sample and without more than one supporting read in ancestor samples were retained. (3) Variants were required to have a quality score >30, and be supported by reads mapping to both the plus and minus strands to avoid sequencing artifacts. Subsequently, each candidate mutation was manually examined in the Integrative Genomics Viewer (IGV) [66] to eliminate ambiguous results, including (1) sequencing errors, especially in polymer regions; (2) variants present in ancestor strains but failed to be captured by variant callers; (3) artifacts from spurious alignments. Mutations were annotated in CDS using snpEff(v4.0) to categorize functional impacts [67].

### 2.6. Ligand-Protein Interaction Prediction

The mol2 format files for the 5AzC ligands were obtained from chEBI, and the crystallographic structure of the DIM-2, DIM-5, and DNMT1 receptor proteins were retrieved from Protein Data Bank (https://www.rcsb.org/) (DIM-2 PDB ID:9BAZ; DIM-5 PDB ID:1ML9; DNMT1: 3OS5). After that, ChimeraX 1.10 was used to prepare ligands and receptors, which included removing Het atoms and water molecules, and the addition of polar hydrogen [68]. The protein was examined for any missing residues. Furthermore, Kollman’s charges were used to neutralize protein, and Gasteiger charges were calculated. Prediction of DIM-2, DIM-5, and DNMT1 protein activity pockets through http://www.pkumdl.cn:8000/cavityplus/#/computation, accessed on 20 April 2026. The central xyz axis of the box size (Å) and box center (Å) in DIM-2 protein was 22.0 × 20.0 × 17.0, 141.0 × 126.0 × 117.0. The central xyz axis of the box size (Å) and box center (Å) in DIM-5 protein was 14.5 × 19.5 × 15.0, 23.25 × 55.25 × 31.50. The central xyz axis of the box size (Å) and box center (Å) in DNMT1 protein was 16.5 × 27.5 × 22.5, 53.25 × −40.75 × 25.75. Calculation of the interaction energy was performed with Auto Dock Tools and Vina 4.2 software to predict the interaction between 5AzC and DIM-2 or DIM-5 [69]. The exhaustiveness was set to 8 (default value, which balances sampling accuracy and computational efficiency), and the number of nodes was set to 6 (sufficient for generating reliable binding poses). To ensure the accuracy of docking results, all simulations were performed in triplicate. A RMSD value of less than 2 Å indicates the reliability of the docking pose, following standard practices. Finally, the docked complex was analyzed by using ChimeraX 1.10 to visualize the interactions between the ligands and receptor.

## 3. Results

### 3.1. Genome-Wide Reduction in DNA Methylation and H3K9me3 Marks by 5AzC

To systematically evaluate the impact of the methylation inhibitor 5AzC on both genome-wide DNA methylation and histone methylation in *N. crassa*, we employed WGBS to quantify 5mC and ChIP-seq to measure H3K9me3. These assays were conducted in wild-type strains cultured with and without 5AzC treatment (see Section 2).

**Figure 1:**
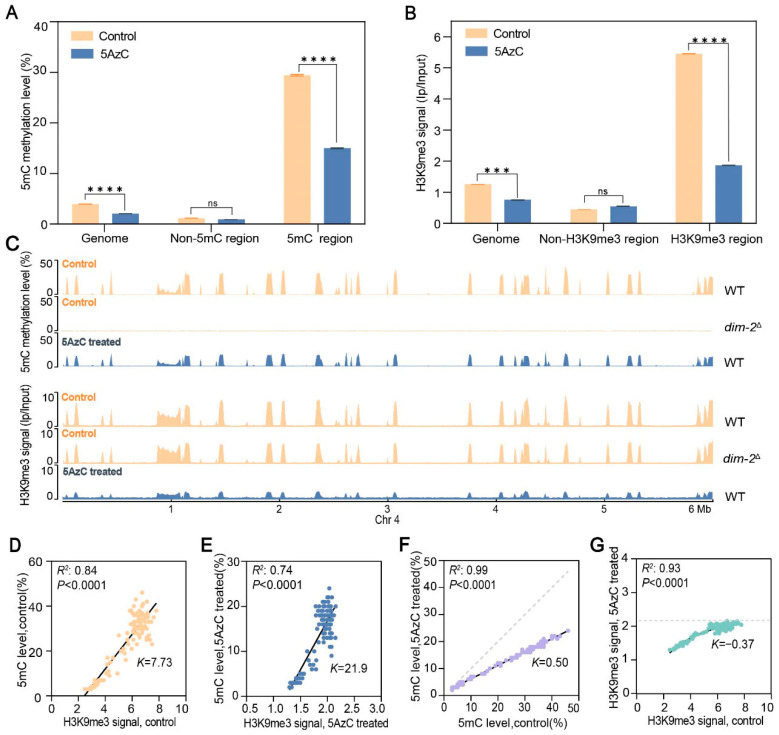
Inhibition of 5mC and H3K9me3 by 5AzC in *N. crassa.* (**A**,**B**) Average DNA methylation levels (**A**) and H3K9me3 signals (**B**) in genome and different regions of WT with or without 5AzC treated. The data are represented by mean ± SEM. *p* values of Mann–Whitney test are shown (****, *p* < 0.0001; ***, *p* < 0.001; ns, *p* > 0.05). (**C**) Patterns of 5mC and H3K9me3 change in Chr4 of WT (with and without 5AzC treated) and *dim-2*^Δ^ mutants. The 5mC and H3K9me3 were calculated as 200 bp sliding windows for the Chr4. H3K9me3 data for *dim-2*^Δ^ was obtained from Basenko [70]. (**D**,**E**) Linear correlation analysis of 5mC and H3K9me3 of WT with (**E**) or without(**D**) 5AzC treated. (**F**) Linear correlation analysis of 5mC in WT with or without 5AzC treatment. (**G**) Nonlinear correlation analysis of H3K9me3 signal in WT strain with or without 5AzC treatment was performed using an exponential decay model. The fitted curve (y = 2.22–2.5e^(−0.37x)) indicates that H3K9me3 levels approach a uniform baseline of ~2.22 upon 5AzC treatment. The horizontal dashed line marks this asymptotic baseline. The coefficient of determination (*R*^2^) and independent variable (*K*) of the correlation is reported.

Compared with untreated strains, which exhibited an average genome-wide 5mC level of 4.3%, 5AzC-treated strains showed a marked reduction to 2.5%, corresponding to a ~50% decrease (Figure 1A). In parallel, 5AzC treatment also led to an ~40% reduction in genome-wide H3K9me3 levels (Figure 1B). By contrast, the *dim-2* knockout strain, characterized by near-complete loss of DNA methylation, showed no substantial decrease in H3K9me3 levels [70] (Figure 1C and Figure S2). These results indicate that 5AzC acts not only as a DNA methylation inhibitor but also independently reduces H3K9me3 levels. Previous studies have shown that in *Neurospora*, H3K9me3 and DNA 5mC strongly co-localize, particularly within repetitive and transposable element-rich genomic regions [53,59,71,72]. Because H3K9me3 recruits HP1, which in turn recruits DIM-2 to mediate DNA methylation, it remains unclear whether 5AzC reduces DNA methylation directly, indirectly via H3K9me3 depletion, or through both mechanisms. Regardless, our data demonstrate a substantial genome-wide impact of 5AzC on both marks. We next investigated whether these reductions occurred uniformly across the genome or preferentially affected specific genomic regions.

Visualization of methylation profiles revealed a global reduction in both 5mC and H3K9me3 in 5AzC-treated strains compared with untreated controls, with approximately 97% overlap in their genomic distribution (Figure 1C and Figure S2). Within overlapping regions, 5mC levels declined from an average of 30.6% in untreated strains to 15.1% after treatment, representing an approximately 50% reduction (Figure 1A). Similarly, H3K9me3 signals declined by roughly 67% from 5.4 to 1.8 (Figure 1B). To determine whether these reductions were coordinated, we assessed the overlap and correlation between the two marks. In untreated WT strains, ~6.11 Mb of genomic regions carried both marks, with strongly correlated intensities (*R*^2^ = 0.84; Figure 1D). In 5AzC-treated strains, co-localization persisted over nearly the same span (~6.05 Mb, ~99% overlap), although the correlation was slightly reduced (*R*^2^ = 0.74; Figure 1E). Notably, ~5.90 Mb of these regions showed concurrent reductions in both marks, representing ~98% of their co-localized distribution. These results indicate that 5AzC exerts a broad inhibitory effect on both 5mC and H3K9me3 without substantially altering their genomic positioning.

To investigate whether the magnitude of 5AzC-induced reduction was related to initial mark abundance, we divided H3K9me3-enriched regions into 118 non-overlapping windows of 50 kb each and compared their 5mC levels before (WT) and after treatment. We observed a near-perfect correlation between pre- and post- treatment 5mC levels (*R*^2^ = 0.99, *p* < 0.0001; Figure 1F), with a regression slope (*K* = 0.50) significantly below unity, indicating that 5AzC proportionally reduced DNA methylation across the genome. In contrast, H3K9me3 intensities in the same regions followed an asymptotic decline (*R*^2^ = 0.93 for nonlinear fit versus *R*^2^ = 0.89 for linear fit; Figure 1G), suggesting that H3K9me3 was reduced toward a uniformly low baseline rather than in proportion to their initial abundance. While the magnitude of reduction differed between the two marks, their correlation remained high following 5AzC treatment (*R*^2^ = 0.74, Figure 1E), albeit weaker than in untreated strains (*R*^2^ = 0.84; Figure 1D). These results highlight nuanced differences in how 5AzC impacts genome-wide H3K9me3 and DNA methylation levels.

### 3.2. Chromatin State Influences the Region-Specific Mutagenic Effects of 5AzC

To systematically investigate the mutagenic effects of 5AzC in *N. crassa*, we carried out mutation accumulation experiments using WT strains cultured with or without 5AzC supplementation. WGS was performed on six 5AzC-treated samples and 20 controls after 18 days of culture (see Section 2 for details). Using a stringent variant-calling and filtering pipeline, we identified 35 point mutations in controls and a striking 3435 point mutations in 5AzC-treated strains, along with seven small insertion/deletion (InDels) mutations detected exclusively in the latter.

**Figure 2:**
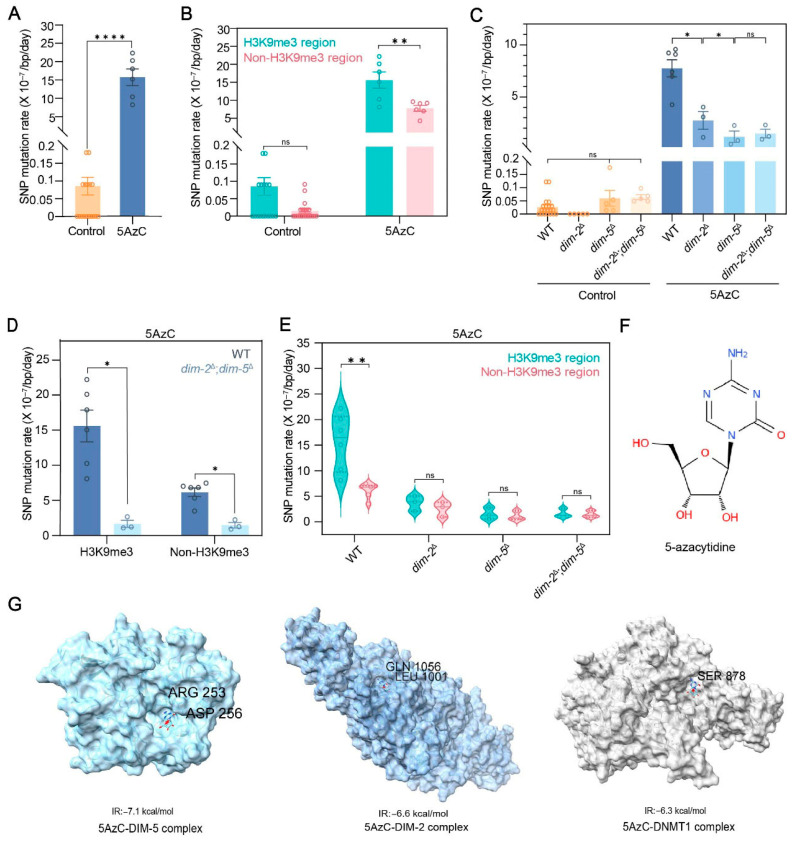
DIM-2 and DIM-5 enhance the mutagenic effects of 5AzC. (**A**) Genomic mutation rate with or without 5AzC treatment. (**B**) Comparison of mutation rates in H3K9me3 and Non-H3K9me3 regions in WT strains with and without 5AzC treatment. (**C**) Estimated SNP mutation rates in WT and mutants (*dim-2*^Δ^, *dim-5*^Δ^, and *dim-2*^Δ^; *dim-5*^Δ^) with or without 5AzC treatment. (**D**) Comparison of mutation rates in H3K9me3 and non-H3K9me3 regions between WT and *dim-2*^Δ^; *dim-5*^Δ^ mutants treated with 5AzC. (**E**) Mutation rate comparison between H3K9me3 and Non-H3K9me3 regions across WT, *dim-2*^Δ^, *dim-5*^Δ^, and *dim-2*^Δ^; *dim-5*^Δ^ mutants with 5AzC treatment. (**F**) The 2D chemical structure of 5-azacytidine (5AzC). (**G**) 3D interactions of 5AzC-protein complex (protein: DIM-2, DIM-5 and DNMT1). IR: interaction energies. Hydrogen bonds were observed in all three complexes, contributing to their stabilization, with the participating amino acids located within the active pocket. The data are represented by mean ± SEM. *p* values of Mann–Whitney test are shown (****, *p* < 0.0001; **, *p* < 0.01; *, *p* < 0.05; ns, *p* > 0.05).

Among the 3435 point mutations induced by 5AzC, C:G->G:C transversions were predominant (~56%), markedly higher than the ~20% observed in controls (Table S2). This mutation spectrum aligns with the C:G->G:C signature previously associated with 5AzC exposure in other organisms, including *E. coli* and HIV-1 [35,36]. The estimated point mutation rates averaged 0.11 per genome per day (2.67 × 10^−9^/bp/day) in controls versus 31.81 per genome per day (7.76 × 10^−7^/bp/day) in 5AzC-treated samples, representing a ~290-fold increase (Figure 2A, Table S3). The dramatic elevation in mutation rate, coupled with the dominance C:G->G:C transversions, underscore the base-specific mutagenic effects of 5AzC in *N. crassa*.

Given our findings that 5AzC affects genome-wide DNA methylation and independently modulates H3K9me3 levels, we next examine whether its mutagenic impact correlates with specific chromatin states, particularly H3K9me3-marked versus unmarked regions. In controls, mutation rates in H3K9me3-marked regions (8.57 × 10^−9^/bp/day) were ~5.8-fold higher than in non-H3K9me3 regions (1.47 × 10^−9^/bp/day; Mann–Whitney U test, *p* = 0.104; Figure 2B, Table S3). In 5AzC-treated samples, mutation rates in H3K9me3 region (1.56 × 10^−6^/bp/day) were ~2.5-fold higher than in non-H3K9me3 regions (6.16 × 10^−7^/bp/day; Mann–Whitney U test, *p* = 0.0022; Figure 2B, Table S3). This pattern indicates that H3K9me3-marked regions consistently accumulate more than unmarked regions, but the relative increase induced by 5AzC is greater in non-H3K9me3 regions.

When comparing 5AzC-treated versus untreated strains within each chromatin state, the highest mutation rate was observed in 5AzC-treated H3K9me3-marked regions, which is approximately 182-fold higher than untreated counterparts. However, while 5AzC-treated non-H3K9me3 regions had ~2.5-fold fewer mutations than treated H3K9me3 regions, they showed the largest relative increase: ~420-fold compared with untreated non-H3K9me3 regions. These results demonstrate that 5AzC markedly elevates mutation rates genome-wide, with a disproportionately strong impact on regions lacking H3K9me3 marks.

### 3.3. DIM-2 and DIM-5 Substantially Enhance Region-Specific Mutagenic Effects of 5AzC

As a methyltransferase inhibitor, 5AzC interacts directly with methyltransferases, impairing their enzymatic activity and altering DNA methylation states across the genome. To investigate whether specific methyltransferases influence the mutagenic effects of 5AzC, we generated *N. crassa* knockout strains lacking DNA methyltransferase DIM-2 (*dim-2*^Δ^), histone methyltransferase DIM-5 (*dim-5*^Δ^) or both (*dim-2*^Δ^; *dim-5*^Δ^) (see Section 2). These strains were cultured with or without 5AzC, followed by whole-genome sequencing to quantify mutation rates.

Under untreated conditions, stringent variant filtering identified 0, 22, and 33 SNPs in *dim-2*^Δ^, *dim-5*^Δ^, and *dim-2*^Δ^; *dim-5*^Δ^ mutants, respectively, corresponding to mutation rates of 0, 5.96 × 10^−9^, and 6.43 × 10^−9^/bp/day, which is comparable to untreated WT strains (2.67 × 10^−9^/bp/day; Mann–Whitney U test, *p* > 0.05; Figure 2C, Tables S2 and S3). Upon 5AzC treatment, mutation rates rose substantially in all knockouts (2.76 × 10^−7^, 1.19 × 10^−7^, and 1.50 × 10^−7^/bp/day for *dim-2*^Δ^, *dim-5*^Δ^, and *dim-2*^Δ^; *dim-5*^Δ^, respectively), but remained 2.8–6.5-fold lower than that in 5AzC-treated WT strains (Mann–Whitney U test, *p* < 0.05; Figure 2C; Table S3). Notably, the double knockout (*dim-2*^Δ^; *dim-5*^Δ^) exhibited only a ~23-fold increase in mutation rate relative to untreated conditions, far less than the ~290-fold increase seen in WT (Figure 2C; Table S3). These results indicate that DIM-2 and DIM-5 together amplify the mutagenic potential of 5AzC by roughly an order of magnitude, suggesting a significant role for these methyltransferases in mediating 5AzC-induced mutagenesis.

Given that DIM-2 and DIM-5 are predominantly enriched in H3K9me3-marked regions, we considered two possible mechanisms: (1) DIM-2 and/or DIM-5 might sequester 5AzC in H3K9me3 regions through reversible protein-drug interactions, increasing local drug concentration and elevating mutation rates predominantly in those regions; (2) DIM-2 and/or DIM-5 might directly enhance the intrinsic mutagenic effect of 5AzC, raising mutation rates across the genome but more strongly in protein-enriched (H3K9me3) regions. The first model (“local enrichment model”) predicts that mutation rates in non-H3K9me3 regions, where DIM-2 and DIM-5 are minimally distributed, would be largely unaffected by 5AzC. In contrast, the second model (“global amplification model”) predicts that non-H3K9me3 regions would also exhibit elevated mutation rates. Both models predict that, in the absence of DIM-2 and DIM-5, 5AzC induced mutation rates should be similar between H3K9me3 and non-H3K9me3 regions.

Our data support the global amplification model. In WT strains, 5AzC-induced mutation rates in H3K9me3-marked regions (1.56 × 10^−6^/bp/day) were ~9.4-fold higher than in *dim-2*^Δ^; *dim-5*^Δ^ double knockouts (1.65 × 10^−7^/bp/day; Mann–Whitney U test, *p* = 0.024; Figure 2D). In non-H3K9me3 regions, WT mutation rates were still ~4.2-fold higher (6.16 × 10^−7^ versus 1.47 × 10^−7^/bp/day; Mann–Whitney U test, *p* = 0.024; Figure 2D). In the absence of both methyltransferases, mutation rates in the two chromatin contexts converged (Mann–Whitney U test, *p* > 0.99; Figure 2E), consistent with a direct DIM-2/DIM-5-5AzC interaction enhancing mutagenicity genome-wide.

To test for direct binding, we performed in silico docking using Auto Dock Tools and Vina 4.2 (Figure 2G; see Section 2), referencing the known covalent interaction between 5AzC and human DNMT1 [17]. Binding affinity predictions (interaction energies) suggested that 5AzC may interact more strongly with DIM-2 and DIM-5 than with DNMT1, with calculated values of −7.1 kcal/mol (DIM-5), −6.6 kcal/mol (DIM-2), and −6.3 kcal/mol (DNMT1) (Figure 2G).

Finally, we asked whether DIM-2 and DIM-5 act independently or synergistically. Both single knockouts (*dim-2*^Δ^ and *dim-5*^Δ^) reduced 5AzC-induced mutation rates (Figure 2C; Table S3), but *dim-5*^Δ^ and *dim-2*^Δ^; *dim-5*^Δ^ strains exhibited similarly low rates, whereas *dim-2*^Δ^ strains retained roughly twice that rate of *dim-5*^Δ^ (Figure 2C; Table S3). This pattern suggests that DIM-5 alone exerts a strong effect, while DIM-2’s contribution partly depends on DIM-5’s presence.

### 3.4. Presence of DIM-2 and DIM-5 Significantly Alters the Mutational Spectrum Induced by 5AzC

**Figure 3:**
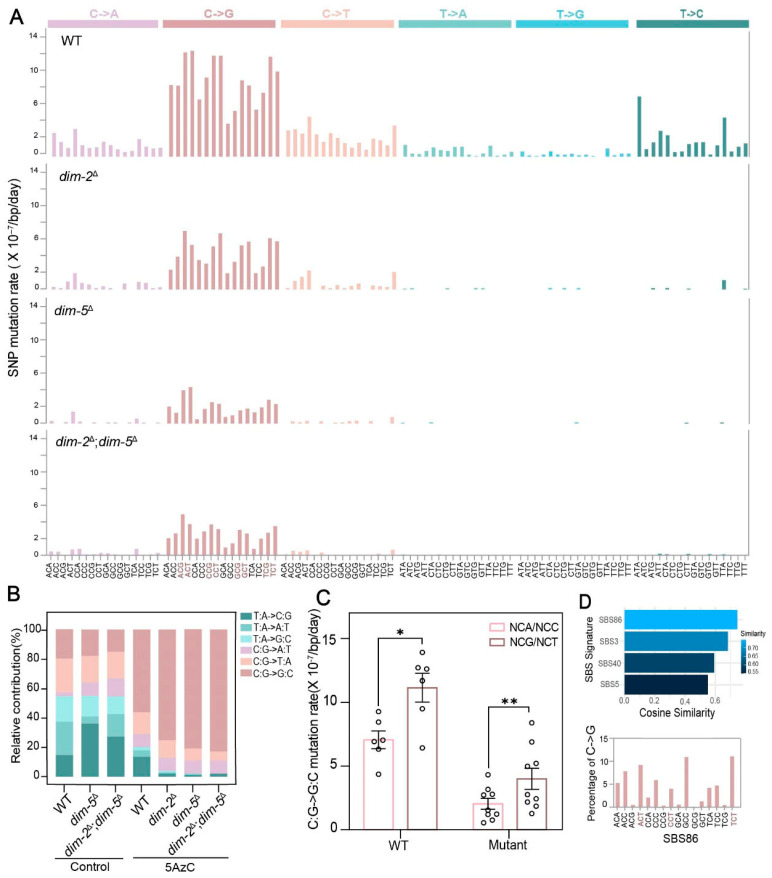
Mutation spectra in WT and mutants after 5AzC treatment. (**A**) SNP rates within each trinucleotide context in WT, *dim-2*^Δ^, *dim-5*^Δ^ and *dim-2*^Δ^; *dim-5*^Δ^ strains with 5AzC treatment. (**B**) Mutation spectrum in WT and mutants with or without 5AzC treatment. (**C**) Comparison of C:G->G:C SNP rates within NCG, NCT contexts and NCA, NCC contexts in WT and mutants with 5AzC treatment. Mutants include *dim-2*^Δ^, *dim-5*^Δ^ and *dim-2*^Δ^; *dim-5*^Δ^ strains. (**D**) Comparison of the WT-5AzC mutational signature to the SBS dataset using cosine similarity analysis. Cosine similarity ranges from 0 (completely distinct) to 1 (identical); values > 0.8 indicate high similarity, 0.7–0.8 moderate similarity, and <0.7 low similarity [77]. The data are represented by mean ± SEM. *p* values of Mann–Whitney test are shown (**, *p* < 0.01; *, *p* < 0.05).

Previous studies on the mutagenic effects of 5AzC, particularly in HIV-1 genomes and specific *E. coli* genes, have reported a strong bias toward mutations at G/C sites (~86.2% in HIV-1), with C:G->G:C transversions being the most frequent (~79.3%) [35]. This preference is thought to arise from 5AzC’s structural similarity to cytidine, which can lead to misincorporation during DNA replication [37,73]. Consistent with these findings, wild-type (WT) *N. crassa* treated with 5AzC also displayed a marked preference for mutations at G/C sites (80.0%; Figure 3B; Table S2), with C:G->G:C transversions accounting for 56.2% of mutations, slightly lower than in HIV and *E. coli*, but still characteristic of 5AzC mutagenesis.

In WT strains treated with 5AzC, classification of mutations into six possible base-change categories revealed a distinctive pattern. The two transition types (C:G->T:A and T:A->C:G) accounted for only 14.8% and 13.4% of mutations, respectively (combined 28.2%; Figure 3B), far below the ~40% typically observed in untreated strains. Among transversion, C:G->G:C changes dominated, representing 56.2% of all mutations (Figure 3B; Table S2). In contrast, *dim-2*^Δ^, *dim-5*^Δ^, and *dim-2*^Δ^; *dim-5*^Δ^ knockout strains treated with 5AzC not only exhibited an ~10-fold reduction in overall mutation rate, but also a striking shift in mutational spectrum. A/T-to-other mutations, which made up ~20% of mutations in WT, were nearly abolished in the mutants (~2.6%; Figure 3B; Table S2). In particular, T:A->C:G transitions dropped from 13.4% in WT to ~1.3% in mutants. Conversely, C:G->G:C transversions increased from 56.2% in WT to an average of 79.7% in the mutants (Figure 3B; Table S2). These observations suggest that DIM-2 and/or DIM-5 strongly potentiate the mutagenic effect of 5AzC, by roughly an order of magnitude, and profoundly reshaped the mutation profile. The near loss of A/T-derived mutations in mutants implies that DIM-2/DIM-5 may interact with specific DNA repair pathways to enhance mutagenesis at A/T sites when present. Moreover, the absence of DIM-2/DIM-5 also led to a significant reduction in C:G->T:A mutations (from 14.8% to 8.5%; Mann–Whitney U test, *p* = 0.011), while it had minimal impact on C:G->A:T mutations (9.0% versus 8.9%; Mann–Whitney U test, *p* = 0.501; Figure 3B and Figure S3; Table S2). This effect is likely because C:G->T:A mutations are primarily driven by deamination of 5-methylcytosine (5mC), whereas C:G->A:T transversions mainly arise from oxidative damage or replication errors that are independent of DNA methylation [74,75].

To further characterize 5AzC-induced mutational signatures, we assessed trinucleotide single-base substitution (SBS) spectra of de novo mutations in WT and mutant strains after treatment (Figure 3A). Within C:G->G:C mutations, C->G substitutions were significantly enriched in NCG and NCT trinucleotide contexts (Repeated Measures (RM) one-way ANOVA, R 4.2.2 with the rstatix package, *p* < 0.05; Figure S4), occurring at rates 1.6–2.0× higher than in NCA and NCC contexts (Figure 3C). This pattern closely resembles (0.75 cosine similarity) the SBS86 mutational signature observed in human cancers (Figure 3D), particularly characterized by enriched C->G substitutions at NCT sites and has been linked to pyrimidine analog chemotherapy in relapsed acute lymphoblastic leukemia (ALL) patients [76].

Additionally, WT strains treated with 5AzC showed pronounced T->C substitution biases in ATA and TTA trinucleotide contexts (Figure 3A). These biases were drastically reduced in *dim-2*^Δ^, *dim-5*^Δ^, and *dim-2*^Δ^; *dim-5*^Δ^ strains (Figure 3A), highlighting the pivotal role of DIM-2 and DIM-5 in shaping both the rate and spectrum of 5AzC-induced mutations.

### 3.5. Elevated Nsy/Syn Ratio Implicates 5AzC-Induced C:G->G:C Transversions in a More Deleterious Mutational Load

To further investigate the impact of 5AzC-induced C:G->G:C transversions on gene function, we analyzed mutations in the coding sequences (CDS) of strains with or without 5AzC treatment using the nonsynonymous-to-synonymous ratio (nsy/syn). The analysis revealed that the nsy/syn ratio for 5AzC-induced C:G->G:C mutations in CDS was 4.97 (939/189; Table 1). In contrast, integration of published mutation data [78] showed that the nsy/syn ratio for SNPs in the CDS of untreated controls was markedly lower, at 2.20 (77/35; Table 1), suggesting that 5AzC-induced C:G->G:C transversions may be deleterious to gene function.

To test this hypothesis, we performed simulation analyses on 1152 C:G->G:C mutations from 5AzC-treated strains and 112 SNPs from untreated strains. To correct for the lower mutation count in the controls, the 112 SNPs were proportionally scaled to 1152 based on the mutational spectrum in CDS. We performed 1000 iterations of random mutagenesis at 1152 sites, simulating both C:G->G:C and SNP introductions. The simulated nsy/syn ratio for C:G->G:C transversions was 4.65 (±0.36), closely matching the empirical value of 4.97 in 5AzC-treated strains (Table 1). In comparison, the simulated nsy/syn ratio for SNPs was 2.60 (±0.17), which aligned with the observed ratio of 2.20 in controls and was significantly lower than that for C:G->G:C transversions in 5AzC-treated strains (Mann–Whitney U test, *p* < 0.0001; Table 1). Together, these simulation results reinforce the conclusion that 5AzC-induced C:G->G:C transversions significantly impair gene function.

### 3.6. Pericentromeric Regions with Low 5mC but High H3K9me3 Exhibit Elevated 5AzC Mutagenicity

**Figure 4:**
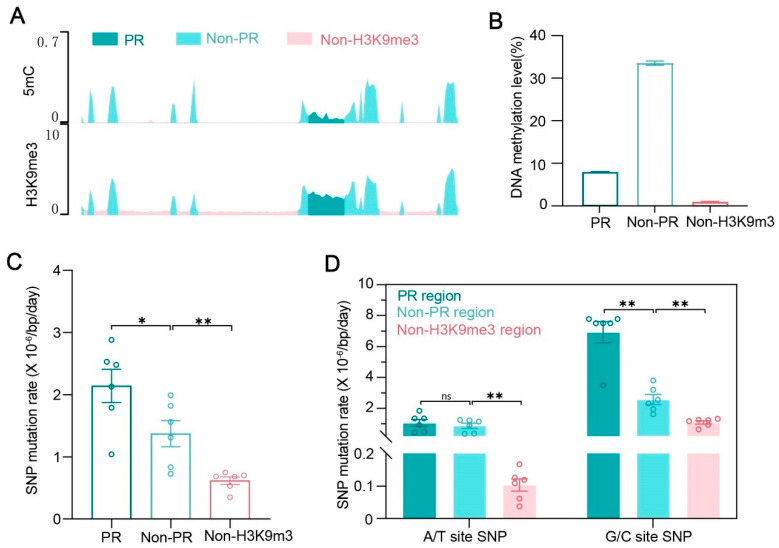
The mutation spectrum in PR and Non-PR regions after 5AzC treatment. (**A**,**B**) The methylation level of 5mC and H3K9me3 in PR, Non-PR, and Non-H3K9me3 regions. PR: pericentromeric regions; Non-PR: Non-PR H3K9me3 regions. (**C**) Comparison the SNP rate in PR, Non-PR regions and Non-H3K9me3 regions. (**D**) Comparison of SNP rate at A/T and G/C site in PR, Non-PR regions, and Non-H3K9me3 regions. The data are represented by mean ± SEM. *p* values of Mann–Whitney test are shown (**, *p* < 0.01; *, *p* < 0.05; ns, *p* > 0.05).

Our analyses reveal that 5AzC induces significantly higher mutation rates in regions marked by H3K9me3 compared to regions lacking this modification. Although H3K9me3-rich regions typically also have high 5mC levels [46,53], we found a notable exception in the pericentromeric regions (PRs) of *N. crassa* [40]. Despite being strongly enriched for H3K9me3, PRs exhibit substantially lower average 5mC content (~8%) compared to other H3K9me3-enriched non-pericentromeric regions (non-PRs, ~33%), though still higher than regions with low H3K9me3 (<1%) (Figure 4A,B).

To investigate how this distinctive epigenetic configuration influences mutagenicity, we subdivided the H3K9me3-enriched regions into PRs and non-PRs by genomic location. In WT strains treated with 5AzC, PRs displayed a mutation rate 1.56-fold higher than non-PRs (2.14 × 10^−6^ versus 1.37 × 10^−6^/bp/day; Unpaired *t* test, *p* = 0.047; Figure 4C; Table S4), despite their lower 5mC content. Moreover, PRs exhibited roughly threefold higher mutation rates than non-H3K9me3 regions (Figure 4C; Table S4).

At A/T sites, mutation rates were nearly identical between PRs (1.06 × 10^−6^/bp/day) and non-PRs (8.66 × 10^−7^/bp/day), contributing 40% and 44% of total mutations, respectively (Mann–Whitney U test, *p* = 0.82; Figure 4D; Table S4). These rates were ~10.3-fold (PR) and ~8.4-fold (non-PR) higher than in non-H3K9me3 regions (1.03 × 10^−7^/bp/day), where A/T mutations accounted for only 8% of total mutations (Figure 4D; Table S4). This indicates that elevated A/T-site mutagenicity is a general feature of H3K9me3-enriched regions after 5AzC treatment, independent of 5mC density.

In contrast, G/C-site mutation rates showed marked regional differences between PRs and non-PRs. PRs exhibited a G/C mutation rate of 6.92 × 10^−6^/bp/day, about 2.7-fold higher than that of non-PRs (2.57 × 10^−6^/bp/day) following 5AzC exposure (Mann–Whitney U test, *p* = 0.0043; Figure 4D; Table S4). This suggests that the genomic context, independent of 5mC density, strongly influences mutation susceptibility. Thus, pericentromeric localization itself enhances vulnerability to 5AzC-induced mutations at G/C sites, highlighting the role of genomic location in shaping mutational dynamics. Moreover, as with A/T sites, the G/C mutation rate in both PRs and non-PRs marked by H3K9me3 was significantly higher than that in non-H3K9me3 regions (1.1 × 10^−6^/bp/day), representing ~6.4-fold and 2.4-fold increases, respectively (Figure 4D; Table S4).

In 5AzC-treated mutants, PRs showed only a modest, statistically non-significant elevation in overall mutation rate (3.15 × 10^−7^/bp/day) compared to non-PRs (2.02 × 10^−7^/bp/day) and non-H3K9me3 regions (1.72 × 10^−7^/bp/day) (Mann–Whitney U test, *p* = 0.13; Figure S5; Table S5). Consistent with the loss of DIM-2/DIM-5, A/T-site mutations were nearly abolished in these mutants (Figure 3A and Figure S5; Table S5). However, G/C-site mutations in PRs persisted, with a significant elevated rate (1.52 × 10^−6^/bp/day) compared to non-PR regions (Mann–Whitney U test, *p* = 0.018), whereas G/C mutation rates did not differ significantly between non-PR (6.33 × 10^−7^/bp/day) and non-H3K9me3 regions (3.25 × 10^−7^/bp/day; Mann–Whitney U test, *p* = 0.136; Figure S5; Table S5).

Collectively, these findings indicate that elevated mutation rates in H3K9me3-marked regions under 5AzC treatment result from two distinct processes: a DIM-2/DIM-5-dependent increase in mutations at both A/T and G/C sites (with a stronger effect at A/T sites), and a pericentromeric-specific enhancement of G/C-site mutations.

## 4. Discussion

5AzC is widely applied in both cancer therapy and epigenetic research as a DNA hypomethylation agent. Consistent with its canonical role, our WGBS analyses revealed a genome-wide reduction of 5mC following treatment, aligning with earlier observations in *N. crassa* [79] and in clinical contexts where 5AzC reactivates tumor suppressor genes [12,13]. However, this demethylation is relatively non-specific: in cancer therapy, it is often accompanied by the reactivation of endogenous retroviral and inhibitory checkpoint molecules (PD-1, PD-L1 and CTLA-4), which can promote drug resistance, immune evasion, and reduced survival [23,24,25,80]. Beyond 5mC loss, we also observed a genome-wide reduction in H3K9me3 after 5AzC treatment. This result cannot be fully explained by DNA demethylation alone, suggesting additional inhibition of KMTs, possibly through activity blocking or protein degeneration [21]. Thus, while 5AzC exerts anticancer effects through demethylation, its broad epigenetic inhibition, including depletion of 5mC and H3K9me3, may also drive adverse outcomes in normal cells [16,26,27]. These findings highlight the need for careful patient stratification and toxicity monitoring clinical practice.

In addition to its epigenetic effects, 5AzC exerts strong mutagenic effects, with a characteristic C:G->G:C transversion bias across diverse systems, including human, mouse, *E. coli*, HIV-1 [31,32,35,36,37,81] (Figure S6). Our WGS data recapitulated this mutational pattern in *N. crassa*, with enrichment of mutations in H3K9me3-marked regions that frequently colocalize with 5mC. Furthermore, constitutive heterochromatin marked by H3K9me3 exhibits reduced chromatin accessibility and undergoes late S-phase replication [82,83]. 5AzC treatment may cause abnormalities in chromatin accessibility and replication timing in these regions, leading to reduced DNA repair efficiency and thereby promoting the enrichment of mutations in H3K9me3-marked regions. Additionally, we also found preferential mutagenesis in PRs, despite their lower average 5mC density compared to non-PRs, suggesting that genomic context (e.g., pericentromeric localization) contributes to mutation susceptibility. Furthermore, 5AzC-induced C->G mutations were enriched in NCT and NCG trinucleotide contexts. While HIV-1 showed enrichment only in NCT and not NCG contexts [35] (Figure S7), we attribute this difference to the lower genomic abundance of NCG motifs in HIV-1 (0.5%) compared to *N. crassa* (2.7%) (Figure S8). Importantly, the 5AzC-induced mutational profile in *N. crassa* displayed moderate similarity to SBS86 (0.75 cosine similarity), a mutation signature identified in relapsed ALL patients following pyrimidine analog chemotherapy [76]. This connection underscores the clinical relevance of our findings.

Our genetic analyses revealed that 5AzC-induced mutagenesis in *N. crassa* depends heavily on DIM-2 and DIM-5. Loss of either enzyme reduced mutagenesis, with DIM-5 playing the dominant role (mediating ~85% of mutations independently) and DIM-2 acting largely in a DIM-5-dependent manner (~64%). This epistatic relationship suggests that DIM-5 and DIM-2 function in the same pathway regulating 5AzC-induced mutagenesis, with DIM-5 acting upstream of DIM-2, potentially through the established H3K9me3–HP1–DIM-2 pathway. These findings parallel observations in other systems: for example, Klaric et al. showed that 5AzC-induced mutagenesis in *E. coli* requires DNMT, while HIV-1 (which lacks DMNT/KMT machinery) shows a restricted spectrum of G/C-only mutations [35,84] (Figure S6), resembling out *dim-2* and/or *dim-5* knockout strains. By contrast, acute myeloid leukemia (AML) samples retain DNMT1 and H3K9 KMT activity, which may explain their broader mutation spectrum, including A/T mutations [31,32,37,81] (Figure S6). Together, these results strongly support a model in which DNMTs and KMTs are not only substrates of 5AzC-mediated inhibition but also critical mediators of its mutagenic effects.

Clinically, 5AzC remains a key therapeutic agent in myeloid malignancies, yet its mutagenic potential raises concerns about treatment resistance and relapse [30,31,32,33]. Recent studies have shown that lysine methyltransferase inhibitors (KMTis) function by competitively blocking the SAM cofactor binding site or disrupting protein–protein interactions (e.g., Menin-KMT2A), thereby inhibiting histone methylation and gene silencing [85]. Our findings raise the possibility that combining 5AzC with KMTis might help reduce mutational burden and improve therapeutic outcomes, although further validation in cancer cell systems is needed. In addition, the strong enrichment of C->G mutations in NCT/NCG motifs may serve as a potential biomarker for 5AzC-induced genomic instability, offering new insights for clinical monitoring of the genetic risks associated with 5AzC therapy. Interestingly, recently studies show that 2′-deoxy-5-azacytidine (DAC), a related DNMTi, induces mitotic defects through DNMT1 activity at methylated DNA [86]. These observations suggest that DNMTs play multifaceted roles in DNMTi response: degradation leads to hypomethylation, while their active presence is required for mutagenesis and mitotic defects. Thus, the clinical application of 5AzC must balance its beneficial demethylating effects against potential risks of genomic instability and acquired mutations, which may depend on the genetic background of tumor [29,33]. Moreover, the 5AzC-induced mutational signature showed moderate similarity to SBS3 (0.7 cosine similarity, Figure 3D), a signature of defective homologous recombination-based repair across multiple tumor types [87,88], further indicates that the mutational impact of 5AzC largely may be modulated by DNA repair status, warranting careful evaluation of patient genotypes before treatment.

In summary, our study systematically evaluated the epigenetic and mutagenic consequences of 5AzC in *N. crassa*. We show that 5AzC induces a global reduction in both 5mC and H3K9me3, accompanied by a substantial mutational burden. While the epigenetic effects stem from the inhibition and/or degeneration of DNMTs and KMTs, the mutagenic effects paradoxically require their presence, especially DIM-5. These findings suggest a potential dual role for DNMTs and KMTs in mediating the therapeutic and mutagenic outcomes of 5AzC. Clinically, our work highlights both the risks of broad epigenetic inhibition and the potential for combination strategies that minimize mutational side effects, offering new insights for optimizing DNMTi-based therapies.

## Figures and Tables

**Table 1:** Comparison of actual and simulated nonsynonymous-to-synonymous ratio (nsy/syn) and mutation counts in the coding sequences (CDS) with or without 5AzC treated.

	Type of Value	Nsy/Syn Ratio	No. of Synonymous Mutations	No. of Nonsynonymous Mutations
**5AzC-treated (C:G->G:C)**	Actual	4.97	189	939
	Simulated	4.65 ± 0.36	204.7 ± 12.94	916.2 ± 13.6
**Control (SNP)**	Actual	2.2	35	77
	Simulated	2.60 ± 0.17	320.7 ± 14.6	763.6 ± 15.4

Simulated values are presented as mean ± SD from 1000 iterations.

## Data Availability

The complete DNA sequencing, 5mC methylation sequencing, and ChIP-seq data generated during this study are available in the National Center for Biotechnology Information Sequence Read Archive repository under study accession number PRJNA1310853 (https://dataview.ncbi.nlm.nih.gov/object/PRJNA1310853?reviewer=un7u11hpm5v1pot3bp03eh0bde, accessed on 20 April 2026). Codes used in this study are available at GitHub (https://github.com/Chenjx25/Epigenetic_analysis, accessed on 20 April 2026). All supplemental figures, tables, and data are available as Supplementary Materials.

## Supplementary Materials

The following supporting information can be downloaded at: https://www.mdpi.com/article/10.3390/jof12050304/s1. The supplementary file includes references that are also listed in the main reference list [31,32,35,70,81] Supplementary File S1: Figure S1. Validation of *dim-2* and *dim-5* gene deletions. Figure S2. Patterns of 5mC and H3K9me3 change in genome of WT, *dim-2*^Δ^ mutants with or without 5AzC treatment. Figure S3. Comparison of the mutation spectrum in WT and mutants after 5AzC treatment. Figure S4. Significance Heatmap of C:G->G:C SNP rates within each trinucleotide context of WT and mutants treated with 5AzC. Figure S5. Comparison the SNP rate, A/T site SNP rate and G/C site SNP rate in PR, Non-PR regions and Non-H3K9me3 regions of 5AzC-treated mutants. Figure S6. The mutation spectrum in *N. crassa* (WT, *dim-2*^Δ^, *dim-5*^Δ^, *dim-2*^Δ^; *dim-5*^Δ^ strains), MDS/AML patient, and HIV-1 with 5AzC treatment. Figure S7. Comparison the proportion of C->G mutation across trinucleotide contexts in *N. crassa* and HIV-1. Figure S8. Trinucleotide content in *N. crassa* and HIV-1. Table S1. Primers used in this study. Table S2. SNP mutation spectrum of WT and mutant strains with or without 5AzC treated. Table S3. SNP mutation rates of WT and mutant strains with or without 5AzC treatment in different regions. Table S4. SNP number and SNP rate at different sites in different regions of WT with 5AzC treatment. Table S5. SNP number and SNP rate at different sites in different regions of mutants with 5AzC treatment. Supplementary File S2: Sequencing sample information and identified mutations.

## Abbreviations

The following abbreviations are used in this manuscript:

5AzC	5-azacytidine
5mC	5-methylcytosine
ChIP	Chromatin immunoprecipitation
DNMT	DNA methyltransferases
DNMTi	DNMT inhibitor
ERV	endogenous retroviruse
H3K9me3	histone H3 lysine 9 trimethylation
DMSO	dimethyl sulfoxide
KMT	lysine methyltransferases
KMTi	lysine methyltransferase inhibitor
MA	mutation accumulation
PRs	pericentromeric regions
WGS	Whole genome resequencing
WGBS	Whole-genome bisulfite sequencing
WT	wild-type
