# Supplementary material for: Epigenetic Inhibitor 5-Azacytidine Triggers DIM-2/DIM-5-Dependent Mutagenesis in H3K9me3-Enriched Regions of Neurospora crassa
Source: J Fungi (Basel). 2026 Apr 22;12(5):304. doi: 10.3390/jof12050304 (PMC13207899; doi:10.3390/jof12050304)
Supplement: Supplementary file 1 [file jof-12-00304-s001.zip › jof-4248914-supplementary/Supplemental Material/Supplemental_Information.pdf]

Supplementary Information

for

Epigenetic Inhibitor 5-Azacytidine Triggers DIM-2/DIM-5–Dependent Mutagenesis in H3K9me3-Enriched Regions of *Neurospora crassa*

Ruonan Yao[1]†, Jingxuan Chen[1]†, Huawei Tan[1], Yile Sun[1], Sihai Yang[1,2], Long Wang[1], Ju Huang[3*], Xiaohui Zhang[1*]

Affiliations:

[1] State Key Laboratory of Pharmaceutical Biotechnology, School of Life Sciences, Nanjing University, Nanjing, Jiangsu 210023, China

[2] Co-Innovation Center for Sustainable Forestry in Southern China, Nanjing Forestry University, Nanjing, Jiangsu 210037, China

[3] State Key Laboratory of Crop Genetics and Germplasm Enhancement, Bioinformatics Center, Academy for Advanced Interdisciplinary Studies, Nanjing Agricultural University, Nanjing 210095, China

† These authors contributed equally

* To whom correspondence should be addressed. Address correspondence to Ju Huang, ponyhuang@njau.edu.cn; Xiaohui Zhang, xiaohuizhang@nju.edu.cn.

SUPPLEMENTARY INFORMATION

Contents

Supplementary Figures

Supplementary Figure S1. Validation of *dim-2* and *dim-5* gene deletions.

Supplementary Figure S2. Patterns of 5mC and H3K9me3 change in genome of WT and *dim-2*$^{\Delta}$ mutants with or without 5AzC treatment.

Supplementary Figure S3. Comparison of the mutation spectrum in WT and mutants after 5AzC treatment.

Supplementary Figure S4. Significance heatmap of C->G SNP rates within each trinucleotide context of WT and mutants treated with 5AzC.

Supplementary Figure S5. Comparison the SNP rate, A/T site SNP rate and G/C site SNP rate in PR, Non-PR regions and Non-H3K9me3 regions of 5AzC-treated mutants.

Supplementary Figure S6. The mutation spectrum in *N. crassa* (WT, *dim-2*$^{\Delta}$, *dim-5*$^{\Delta}$, *dim-2*$^{\Delta}$;*dim-5*$^{\Delta}$ strains), MDS/AML patient, and HIV-1 with 5AzC treatment.

Supplementary Figure S7. Comparison the proportion of C->G mutation across trinucleotide contexts in *N. crassa* and HIV-1.

Supplementary Figure S8. Trinucleotide content in *N. crassa* and HIV-1.

Supplementary Tables

Supplementary Table S1. Primers used in this study.

Supplementary Table S2. SNP mutation spectrum of WT and mutant strains with or without 5AzC treated.

Supplementary Table S3. SNP mutation rates of WT and mutant strains with or without 5AzC treatment in different regions.

Supplementary Table S4. SNP number and SNP rate at different site in different regions of WT with 5AzC treatment.

Supplementary Table S5. SNP number and SNP rate at different site in different regions of mutants with 5AzC treatment.

Reference

Supplementary Figures

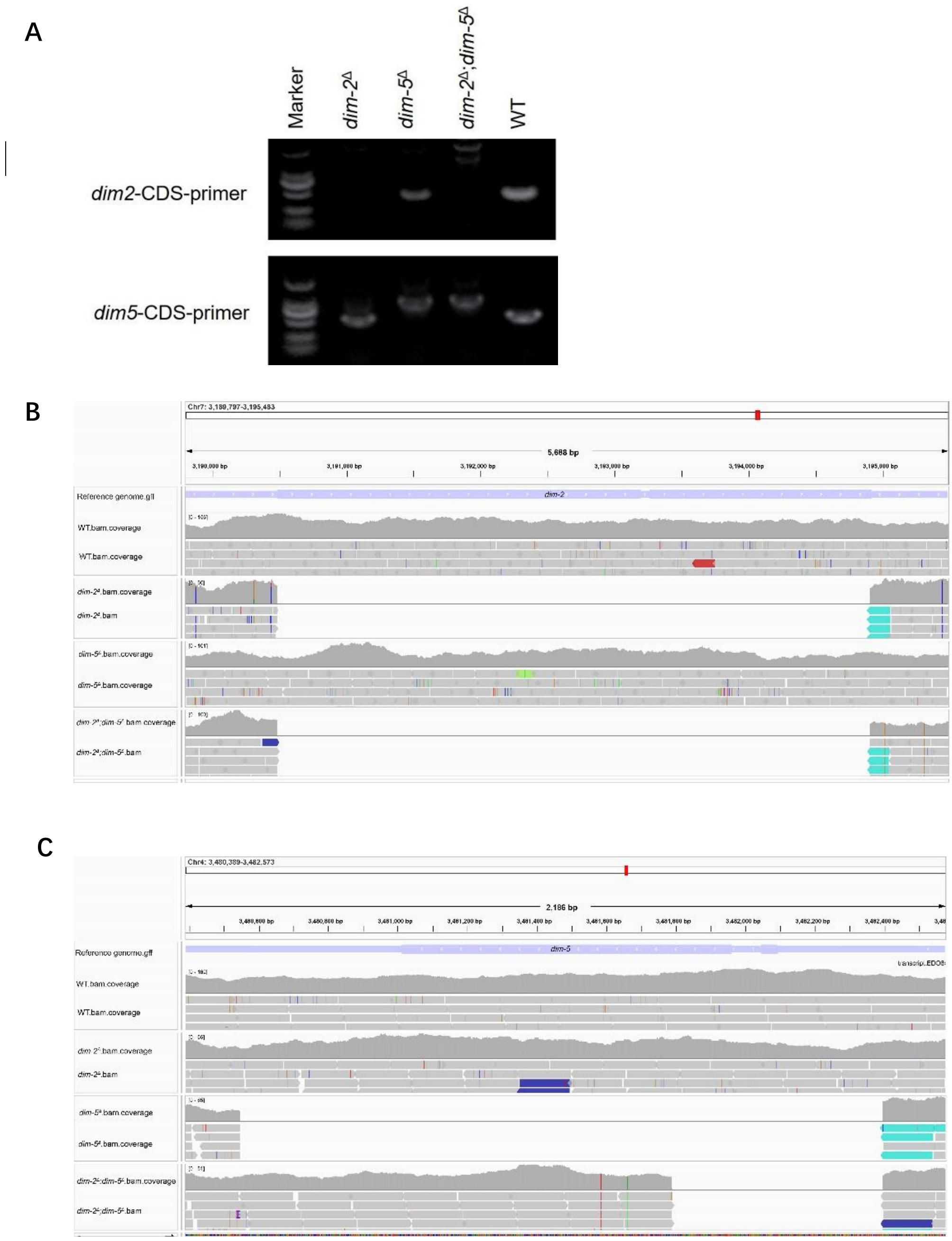

Supplementary Figure S1. Validation of *dim-2* and *dim*-5 gene deletions. (A) PCR validation. CDS-specific primers for *dim-2* and *dim-5* were performed on genomic DNA from WT, *dim-2*$^{\Delta}$, *dim-5*$^{\Delta}$, and *dim-2*$^{\Delta}$;*dim-5*$^{\Delta}$ strains. Absence of bands in the mutant strains confirms reading frame disruption; WT shows expected amplicons. (B, C) IGV snapshots of WGS data at *dim-2* (B) and *dim-5* (C) loci. Reads cover the gene loci in WT but are absent or reduced in knockout strains. In the *dim-2*$^{\Delta}$;*dim-5*$^{\Delta}$ strain, the *dim-5* locus retains a partial deletion spanning from positions 3,481,786 to 3,482,390, which removes the promoter and a portion of the CDS, leading to disruption of the reading frame. Genomic coordinates: *dim-2* (Chr.7: 3,189,797–3,195,483); *dim-5* (Chr.4: 3,480,589–3,482,273).

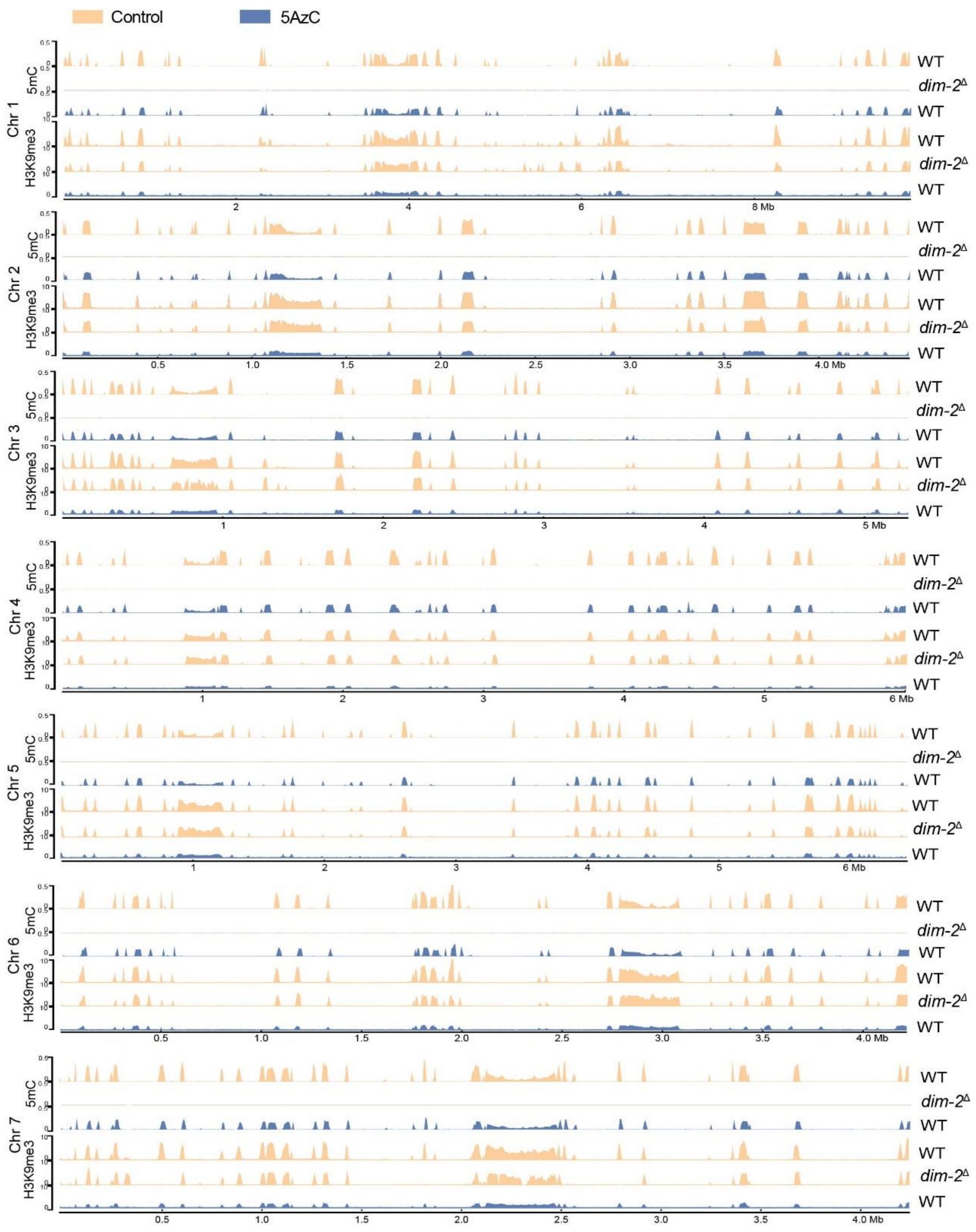

Supplementary Figure S2. Patterns of 5mC and H3K9me3 change in genome of WT, *dim-2*$^{\Delta}$ mutants with or without 5AzC treatment. The 5mC and H3K9me3 was calculated as 200bp windows for the chromosome. H3K9me3 data for *dim-2*$^{\Delta}$ was obtained from Basenko(1).

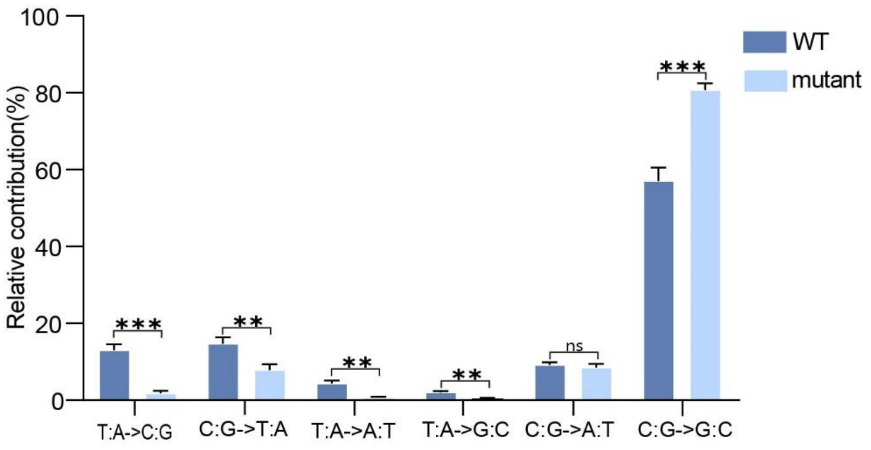

Supplementary Figure S3. Comparison of the mutation spectrum in WT and mutants after 5AzC treatment. Mutants including *dim-2*$^{\Delta}$, *dim-5*$^{\Delta}$and *dim-2*$^{\Delta}$;*dim-5*$^{\Delta}$ strains. *P* values of Mann Whitney test are shown (****, *P* < 0.0001; **, *P* < 0.01; *, *P* < 0.05; ns, *P* > 0.05).

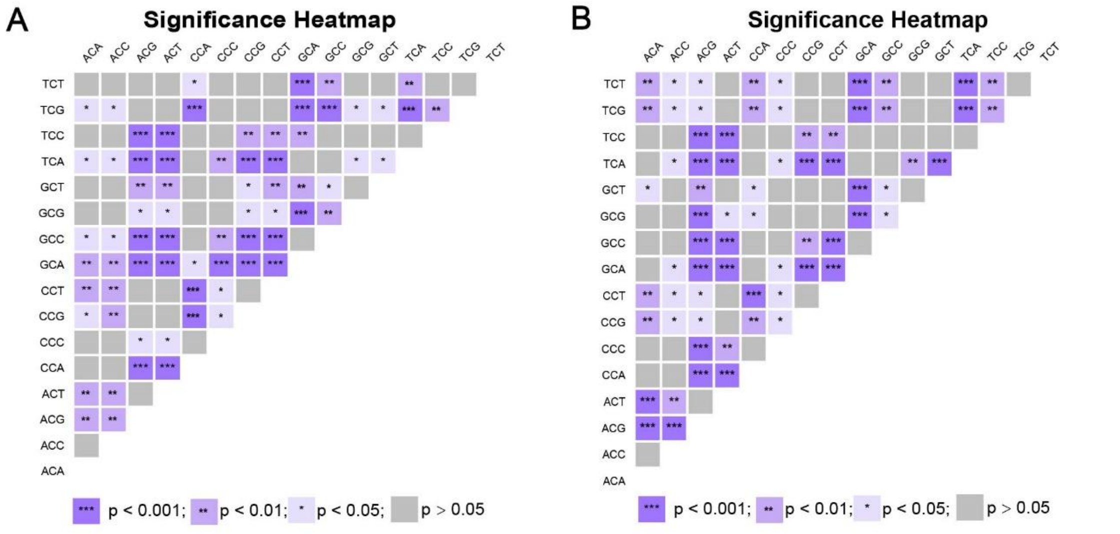

Supplementary Figure S4. Significance heatmap of C->G SNP rates within each trinucleotide context of WT **(A)** and mutants **(B)** treated with 5AzC. Mutants including *dim-2*$^{\Delta}$, *dim-5*$^{\Delta}$and *dim-2*$^{\Delta}$;*dim-5*$^{\Delta}$ strains. P values of Repeated Measures (RM) one-way ANOVA are shown (***, $P < 0.001$; **, $P < 0.01$; *, $P < 0.05$; ns, $P > 0.05$).

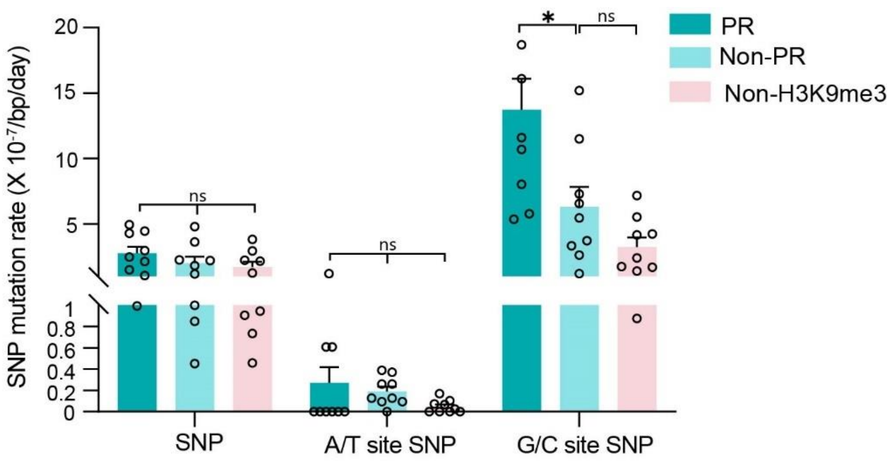

Supplementary Figure S5. Comparison the SNP rate, A/T site SNP rate and G/C site SNP rate in PR, Non-PR regions and Non-H3K9me3 regions of 5AzC-treated mutants. Mutants including *dim-2*$^{\Delta}$, *dim-5*$^{\Delta}$and *dim-2*$^{\Delta}$;*dim-5*$^{\Delta}$ strains. *P* values of Mann Whitney test are shown (*, $P < 0.05$; ns, $P > 0.05$).

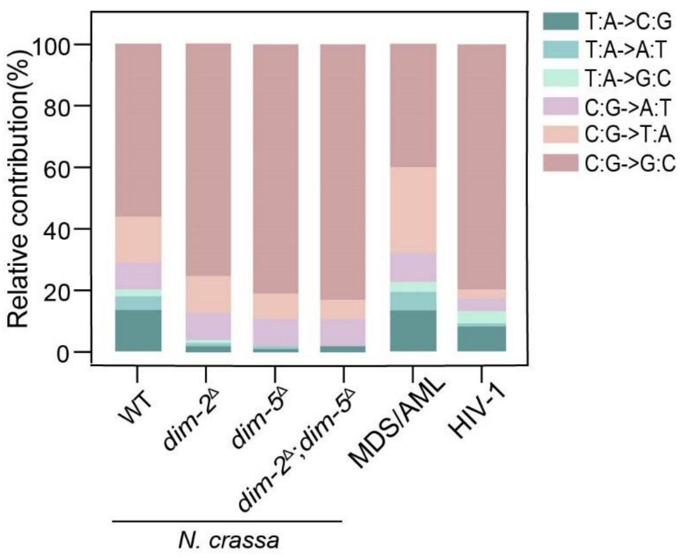

Supplementary Figure S6. The mutation spectrum in *N. crassa* (WT, *dim-2*△, *dim-5*△, *dim-2*△;*dim-5*△ strains), MDS/AML patient(2–4), and HIV-1(5) with 5AzC treatment. MDS/AML: myelodysplastic syndromes (MDS) and older adults with acute myeloid leukemia (AML). The mutagenesis experiment of 5AzC on HIV-1 was conducted in human U373-MAGI-CXCR4CEM cells.

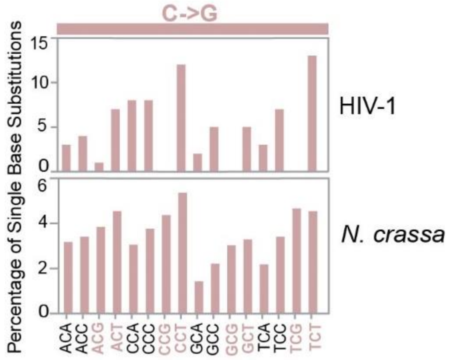

Supplementary Figure S7. Comparison the proportion of C->G mutation across trinucleotide contexts in *N. crassa* and HIV-1(6).

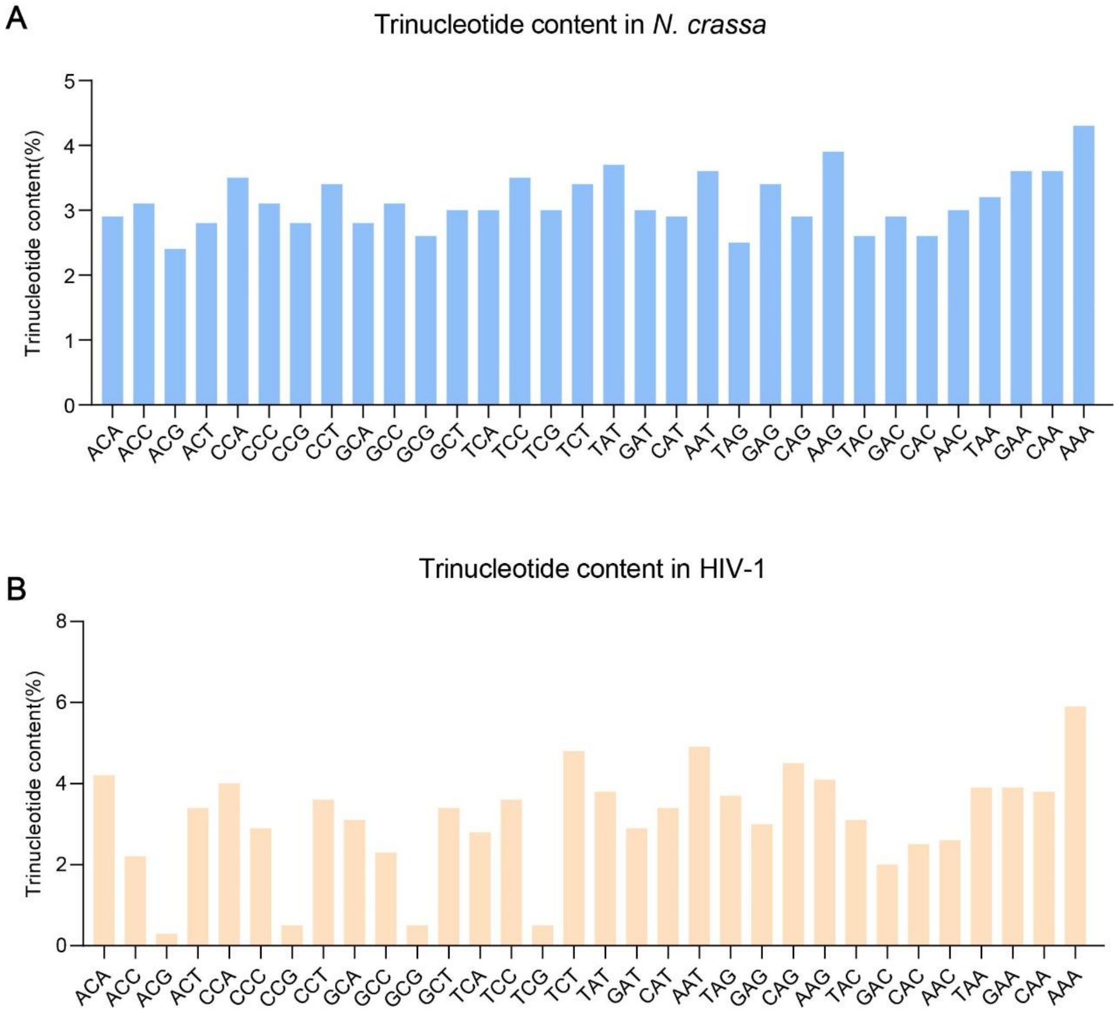

Supplementary Figure S8. Trinucleotide content in *N. crassa* **(A)** and HIV-1 **(B)**.

Supplementary Tables

Supplementary Table S1. Primers used in this study.

Primer	Sequence(5'-3')
**a. For *dim-2*-knockout**	
dim-2-5flankF	CACACAGAACCCTAAAGAAAC
dim-2-5flankR	CCCAAGCTTGTCTGGCTGATAACGAACT
dim-2-3flankF	CGGGATCCGTTGTAGTGCGACGAGGTA
dim-2-3flankR	TGCCCTGCTAACAAAGAC
dim-2-hphF	TCGTTATCAGCCAAGCAAGCTTGGGGAAAGGCGGACAGGTATC
dim-2-hphR	TCGTCGCACTACAACGGATCCCGTTCTGGGTAAACGACCTATAG
**b. For *dim-5*-knockout**	
dim-5-5flankF	CGTTTCCGCACAATGTC
dim-5-5flankR	ACATCTTGACGGTTTGGTGCTCTAGAGCCTTCCGAGTAGGCTGCT
dim-5-3flankF	CTCACTCAAAGGCGGTAATAGCTCTAGAGCGGTGTCACAGGAATGGAA
dim-5-3flankR	TATCAGCCCATTGCGAGTG
dim-5-barF	GCTCTAGAGCCACAAACCGTCAAAGATGT
dim-5-barR	GCTCTAGAGCTATTACCGCTTTGAGTGAG
**c. PCR validation of *dim-2* and *dim-5* gene deletions**	
dim2-CDS-primer	
dim2-CDS-F	AACTATGCTTACCACCCTCAA
dim2-CDS-R	TTCACAACTTCATCCACACG
dim5-CDS-primer	
dim5-CDS-F	CGACGACCCTCAACTTCA
dim5-CDS-R	AGCACCTTGGTCGCTACA

Supplementary Table S2. SNP mutation spectrum of WT and mutant strains with or without 5AzC treated. Values outside (inside) parentheses denote mutation counts (frequency, %)

		A:T->G:C	C:G->T:A	A:T->T:A	A:T->C:G	C:G->A:T	C:G->G:C	Total
**Control**	**WT**	5(14.3%)	8(22.9%)	8(22.9%)	6(17.1%)	1(2.9%)	7(20.0%)	35
	dim-2$^{\Delta}$	0	0	0	0	0	0	0
	dim-5$^{\Delta}$	8(36.4%)	4(18.2%)	1(4.5%)	3(13.6%)	2(9.1%)	4(18.2%)	22
	dim-2$^{\Delta}$**;*dim-5***$^{\Delta}$	9(27.3%)	6(18.2%)	5(15.2%)	4(12.1%)	4(12.1%)	5(15.2%)	33
**5AzC treated**	**WT**	460(13.4%)	508(14.8%)	155(4.5%)	73(2.1%)	307(8.9%)	1932(56.2%)	3435
	dim-2$^{\Delta}$	11(1.8%)	72(11.8%)	5(0.8%)	4(0.7%)	54(8.8%)	465(76.1%)	611
	dim-5$^{\Delta}$	2(0.8%)	21(8.0%)	2(0.8%)	1(0.4%)	24(9.1%)	214(81.1%)	264
	dim-2$^{\Delta}$**;*dim-5***$^{\Delta}$	7(1.5%)	26(5.6%)	2(0.4%)	1(0.2%)	41(8.9%)	384(83.3%)	461

Supplementary Table S3. SNP mutation rates of WT and mutant strains with or without 5AzC treatment in different regions. The data are represented by mean ± SEM.

	Strains	Genome		H3K9me3 region		Non-H3K9me3 region	
		SNP number (/sample/day)	SNP rate ($\times 10^{-7}$ /bp/day)	SNP number (/sample/day)	SNP rate ($\times 10^{-7}$ /bp/day)	SNP number (/sample/day)	SNP rate ($\times 10^{-7}$ /bp/day)
**Control**	**WT**	0.11(±0.03)	0.027(±0.008)	0.06(±0.02)	0.086(±0.025)	0.05(±0.02)	0.015(±0.006)
	dim-2$^{\Delta}$	0	0	0	0	0	0
	dim-5$^{\Delta}$	0.24(±0.12)	0.059(±0.031)	0.11(±0.09)	0.16 (±0.12)	0.13(±0.04)	0.039(±1.22)
	dim-2$^{\Delta}$**;*dim-5***$^{\Delta}$	0.26(±0.04)	0.064 (±0.009)	0.19(±0.05)	0.277(±0.07)	0.07(±0.01)	0.021(±0.004)
**5AzC treated**	**WT-5AzC**	31.81(±3.36)	7.76(±0.82)	11.86(±1.44)	15.60(±2.20)	22.81(±1.23)	6.16(±0.61)
	dim-2$^{\Delta}$***-5AzC***	11.31(±3.50)	2.76(±0.86)	2.56(±0.60)	3.69(±0.87)	8.76(±2.91)	2.57(±0.85)
	dim-5$^{\Delta}$***-5AzC***	4.89(±2.26)	1.19(±0.55)	1.07(±0.46)	1.55(±0.66)	3.81(±1.81)	1.12(±0.53)
	dim-2$^{\Delta}$**;*dim-5***$^{\Delta}$***-5AzC***	6.15(±1.70)	1.50(±0.41)	1.15(±0.35)	1.65(±0.50)	5.00(±1.36)	1.47(±0.40)

Supplementary Table S4. SNP number and SNP rate at different sites in different regions of WT with 5AzC treatment. The data are represented by mean ± SEM.

WT-5AzC	PR region		Non-PR rgion		Non-H3K9me3 region	
	SNP number	SNP rate ($\times 10^{-7}$/bp/day)	SNP number	SNP rate ($\times 10^{-7}$/bp/day)	SNP number	SNP rate ($\times 10^{-7}$/bp/day)
**A:T->G:C**	64(±2.30)	6.49(±1.40)	281(±9.42)	6.05(±1.22)	115(±3.53)	0.65(±0.12)
**A:T->T:A**	26(±1.67)	2.64(±1.01)	84(±3.36)	1.81(±0.43)	45(±1.88)	0.26(±0.06)
**A:T->C:G**	14(±0.71)	1.42(±0.44)	37(±1.42)	0.80(±0.18)	22(±0.99)	0.13(±0.03)
**A/T site**	**104(±3.67)**	**10.55(±2.23)**	**402(±13.08)**	**8.66(±1.69)**	**182(±5.61)**	**1.03(±0.19)**
**C:G->A:T**	17(±0.48)	7.59(±1.28)	85(±2.18)	4.30(±0.66)	205(±4.28)	1.07(±013)
**C:G->T:A**	49(±1.01)	21.89(±2.72)	149(±4.73)	7.54(±1.44)	310(±9.81)	1.61(±0.31)
**C:G->G:C**	89(±1.72)	39.72(±4.61)	273(±4.94)	13.82(±1.50)	1570(±27.67)	8.17(±0.86)
**G/C site**	**155(±2.57)**	**69.18(±6.89)**	**507(±10.87)**	**25.66(±3.30)**	**2085(±34.01)**	**10.86(±1.06)**
**Total**	**259(±5.39)**	**21.41(±2.67)**	**909(±23.18)**	**13.74(±2.10)**	**2267(±37.40)**	**6.16(±0.61)**

Supplementary Table S5. SNP number and SNP rate at different sites in different regions of mutants with 5AzC treatment. Mutants including *dim-2*$^{\Delta}$, *dim-5*$^{\Delta}$and *dim-2*$^{\Delta}$;*dim-5*$^{\Delta}$ strains. The data are represented by mean ± SEM.

mutant-5AzC	PR region		Non-PR region		Non-H3K9me3 region	
	SNP number	SNP rate ($\times10^{-7}$/bp/day)	SNP number	SNP rate ($\times10^{-7}$/bp/day)	SNP number	SNP rate ($\times10^{-7}$/bp/day)
**A:T->G:C**	2(±0.17)	0.14(±0.09)	13(±0.63)	0.17(±0.05)	5(±0.33)	0.02(±0.009)
**A:T->T:A**	2(±0.17)	0.14(±0.09)	1(±0.17)	0.01(±0.01)	6(±0.21)	0.02(±0.008)
**A:T->C:G**	2(±0.17)	0.14(±0.09)	1(±0.17)	0.01(±0.01)	3(±0)	0.01(±0.006)
**A/T site**	**6(±0.24)**	**0.41(±0.14)**	**15(±0.41)**	**0.19(±0.04)**	**14(±0.49)**	**0.05(±0.02)**
**C:G->A:T**	10(±0.45)	2.81(±0.84)	19(±0.37)	0.59(±0.12)	90(±2.5)	0.28(±0.07)
**C:G->T:A**	17(±0.76)	4.56(±1.28)	31(±1.11)	0.96(±0.26)	71(±0.88)	0.24(±0.09)
**C:G->G:C**	30(±0.79)	7.84(±1.16)	154(±2.85)	4.77(±1.21)	879(±21.4)	2.73(±0.57)
**G/C site**	**57(±1.13)**	**15.2(±2.67)**	**204(±4.84)**	**6.33(±1.50)**	**1040(±24.21)**	**3.25(±0.72)**
**Total**	**63(±1.61)**	**3.15(±0.53)**	**219(±4.27)**	**2.02(±0.47)**	**1054(24.56)**	**1.72(±0.38)**

References

1. Basenko EY, Sasaki T, Ji L, Prybol CJ, Burckhardt RM, Schmitz RJ, Lewis ZA. 2015. Genome-wide redistribution of H3K27me3 is linked to genotoxic stress and defective growth. Proc Natl Acad Sci U S A 112: E6339-6348.

2. Walter MJ, Shen D, Ding L, Shao J, Koboldt DC, Chen K, Larson DE, McLellan MD, Dooling D, Abbott R, Fulton R, Magrini V, Schmidt H, Kalicki-Veizer J, O'Laughlin M, Fan X, Grillot M, Witowski S, Heath S, Frater JL, Eades W, Tomasson M, Westervelt P, DiPersio JF, Link DC, Mardis ER, Ley TJ, Wilson RK, Graubert TA. 2012. Clonal architecture of secondary acute myeloid leukemia. N Engl J Med 366:1090–1098.

3. Uy GL, Duncavage EJ, Chang GS, Jacoby MA, Miller CA, Shao J, Heath S, Elliott K, Reineck T, Fulton RS, Fronick CC, O'Laughlin M, Ganel L, Abboud CN, Cashen AF, DiPersio JF, Wilson RK, Link DC, Welch JS, Ley TJ, Graubert TA, Westervelt P, Walter MJ. 2017. Dynamic changes in the clonal structure of MDS and AML in response to epigenetic therapy. Leukemia 31:872–881.

4. Welch JS, Petti AA, Miller CA, Fronick CC, O'Laughlin M, Fulton RS, Wilson RK, Baty JD, Duncavage EJ, Tandon B, Lee Y-S, Wartman LD, Uy GL, Ghobadi A, Tomasson MH, Pusic I, Romee R, Fehniger TA, Stockerl-Goldstein KE, Vij R, Oh ST, Abboud CN, Cashen AF, Schroeder MA, Jacoby MA, Heath SE, Luber K, Janke MR, Hantel A, Khan N, Sukhanova MJ, Knoebel RW, Stock W, Graubert TA, Walter MJ, Westervelt P, Link DC, DiPersio JF, Ley TJ. 2016. TP53 and Decitabine in Acute Myeloid Leukemia and Myelodysplastic Syndromes. N Engl J Med 375:2023–2036.

5. Rawson JMO, Daly MB, Xie J, Clouser CL, Landman SR, Reilly CS, Bonnac L, Kim B, Patterson SE, Mansky LM. 2016. 5-Azacytidine Enhances the Mutagenesis of HIV-1 by Reduction to 5-Aza-2’-Deoxycytidine. Antimicrob Agents Chemother 60:2318–2325.

6. Rawson JMO, Daly MB, Xie J, Clouser CL, Landman SR, Reilly CS, Bonnac L, Kim B, Patterson SE, Mansky LM. 2016. 5-Azacytidine Enhances the Mutagenesis of HIV-1 by Reduction to 5-Aza-2’-Deoxycytidine. Antimicrob Agents Chemother 60:2318–2325.
